# Atomic-level structural correlations across the morphotropic phase boundary of a ferroelectric solid solution: *x*BiMg_1/2_Ti_1/2_O_3_-(1 − *x*)PbTiO_3_

**DOI:** 10.1038/s41598-017-00530-z

**Published:** 2017-03-28

**Authors:** Kaustuv Datta, Reinhard B. Neder, Jun Chen, Joerg C. Neuefeind, Boriana Mihailova

**Affiliations:** 1 Department of Earth Sciences, University of Hamburg, Hamburg, D-20146 Germany; 2 Department of Crystallography and Structure Physics, University of Erlangen-Nürnberg, Staudtstraße 3, Erlangen, 91058 Germany; 3 School of Metallurgical and Ecological Engineering, University of Science and Technology Beijing, Beijing, China; 4 Chemical and Engineering Materials Division, Oak Ridge National Laboratory, Oak Ridge, Tennessee 37831 USA

## Abstract

Revelation of unequivocal structural information at the atomic level for complex systems is uniquely important for deeper and generic understanding of the structure property connections and a key challenge in materials science. Here we report an experimental study of the local structure by applying total elastic scattering and Raman scattering analyses to an important non-relaxor ferroelectric solid solution exhibiting the so-called composition-induced morphotropic phase boundary (MPB), where concomitant enhancement of physical properties have been detected. The powerful combination of static and dynamic structural probes enabled us to derive direct correspondence between the atomic-level structural correlations and reported properties. The atomic pair distribution functions obtained from the neutron total scattering experiments were analysed through big-box atom-modelling implementing reverse Monte Carlo method, from which distributions of magnitudes and directions of off-centred cationic displacements were extracted. We found that an enhanced randomness of the displacement-directions for all ferroelectrically active cations combined with a strong dynamical coupling between the A- and B-site cations of the perovskite structure, can explain the abrupt amplification of piezoelectric response of the system near MPB. Altogether this provides a more fundamental basis in inferring structure-property connections in similar systems including important implications in designing novel and bespoke materials.

## Introduction

Ferroelectric materials with a composition-driven structural crossover, commonly known as morphotropic phase boundary (MPB), have become an indispensable part of many modern devices, particularly used as sensors, actuators and memories, utilizing their superior properties at the MPB. The term MPB, which literally refers to a boundary between two forms, was first coined to describe the chemically induced change in the ferroelectric long-range order of the famous PbZr_*x*_Ti_1−*x*_O_3_ (PZT). Since then, it is now a well established fact that invoking a structural instability by tweaking the composition may result in anomalous characteristics similar to PZT. The current understanding of an MPB and the associated enhancement of certain physical properties of a ferroelectric material primarily relies on the fact that the system acquires a state where the rotation of the unit-cell polarization vector becomes easier due to the development of additional degrees of freedom either in a single low-symmetry phase or in several coexisting phases. The concept of a bridging low-symmetry phase became noted after the discovery of a monoclinic *Cm* phase at the MPB of PZT^1–3^ and manifested a renewed interest in studying as well as in designing bespoke complex ferroelectric materials. However in recent years it has also been demonstrated that the analysis of the gross average structure broadly simplifies the ubiquitous and varied complexity of the mesoscopic-scale structural features, which are crucial to understand the occurrence of anomalous properties at the MPB^4–10^. Especially, with many competing interactions and structural frustrations at the atomic level, multi-component ferroelectric materials in general provide a unique challenge in developing precise structural models that would correspond to the physical properties^11^. Consequently, it is still a riveting topic to investigate aspects of structural behaviour even for immensely studied systems like PZT, in search of a more rigorous model than the existing concept of easy rotation of the net polarization^12–14^. It is also highly anticipated that the understanding would not be complete unless the static structural models are equally complemented by dynamical information, typically obtainable from inelastic scattering processes^15–18^.

Driven by the motivation of finding Pb-free or reduced-Pb alternatives of PZT, there has been a strong interest in Bi-containing ferroelectric solid solutions with the general formula *x*BiMeO_3_-(1 − *x*)PbTiO_3_, Me = Sc, Fe, Mg_1/2_Ti_1/2_, Ni_1/2_Ti_1/2_, Ni_1/2_Zr_1/2_ etc following the revelation of the MPB features in those systems^19, 20^. For example, *x*BiScO_3_-(1 − *x*)PbTiO_3_ (BS-PT) exhibited even superior physical properties with a higher operational range of temperature than PZT^21, 22^. The successful partial substitution of Pb by Bi without compromising cherished propertied of pure-Pb containing systems was considered as an important milestone for developing eco-friendly materials. Therefore BS-PT, in the form of ceramics, thin films as well as single crystals with desirable properties, gained rapid interest as a potential replacement of PZT. However the high price of Sc_2_O_3_ is making this particular system less accessible. Although not as attractive as BS-PT, *x*BiMg_1/2_Ti_1/2_O_3_-(1 − x)PbTiO_3_ (*x*BMT-PT), first reported in 2004, provides a reasonable alternative to the expensive Sc with promising MPB-properties at *x* ≈ 0.63^23^. It is also considered as an interesting system as BMT was reported to be a structural analogue of anti-ferroelectric PbZrO_3_^24^. In addition, *x*BMT-PT has also been uniquely shown to possess zero thermal expansion coefficient in the range 0.2 ≤ *x* ≤ 0.4, and highly stable piezoelectric properties at non-ambient temperatures^25, 26^. In terms of the structural phase transition driven by the composition, very recently Upadhyay *et al*.^27^ proposed that there is a tetragonal (*P*4 *mm*) to monoclinic (*P* *m*) phase transition through a mixed phase (*P*4 *mm* + *P* *m*) region that exists in the range 0.60 ≤ *x* ≤ 0.67, based on the Rietveld refinements of the powder XRD pattern.

Morphotropic phase boundary in ferroelectric solid solutions has so far been detected through the typical average structural investigation, mostly applying standard powder diffraction technique for perovskite based oxide systems. Although there have been rigorous attempts to study particularly the local structural correlations, such as diffuse scattering studies on Pb-based complex systems revealing strong evidence for large deviations from the average structure^8–10, 28–30^, there is still lack of an experimentally conceived model at the atomic level to identify and correlate the properties to the different facets of the structure, which can then serve as a more fundamental basis in finding and designing superior as well as eco-friendly materials. Hence, there is a pressing need to elucidate mesoscopic-scale atomic correlations in ferroelectric solid solutions to better understand the physics of MPB.

With the present availability of high energy x-ray synchrotron facilities and spallation neutron sources, it is nowadays possible to obtain data for a wide range of reciprocal-lattice vectors Q for powder samples, and thereby to consider Fourier transformation of the data, taking both Bragg diffraction and diffuse scattering on equal weight. This is known as total scattering method which manifests pair distribution functions (PDFs). PDFs essentially describe the whole structure in terms of atom-atom distances weighted by their scattering power. Hence PDFs are critically sensitive to variations of the local correlations and are considered categorically as a powerful local probe^31^. The total scattering method has already been applied to a number of popular ferroelectric systems including both Pb-based and Pb-free compounds, and revealed hitherto unseen structural characteristics, such as large and persistent static displacements of cations from their crystallographic sites, distinct local and average polarisation, chemical ordering, formation of polar nano-regions as well as their developments with composition and temperature^4, 5, 11, 13, 17, 32–37^.

In this report we show the composition-driven evolution of the local cation-environment in *x*BMT-PT as well as their dynamical behaviour through a combined analysis of neutron PDFs and Raman scattering data at ambient conditions for the compounds covering the whole composition-range of stability across the phase diagram, which has not been reported so far. Our experimental results provide deeper insights into the structural phenomena occurring at the MPB of a perovskite-type ferroelectric solid solution and help to establish a comprehensive structure-property relationship for a broad range of systems.

## Results and Discussion

### Pair distribution function analysis

**Figure 1:**
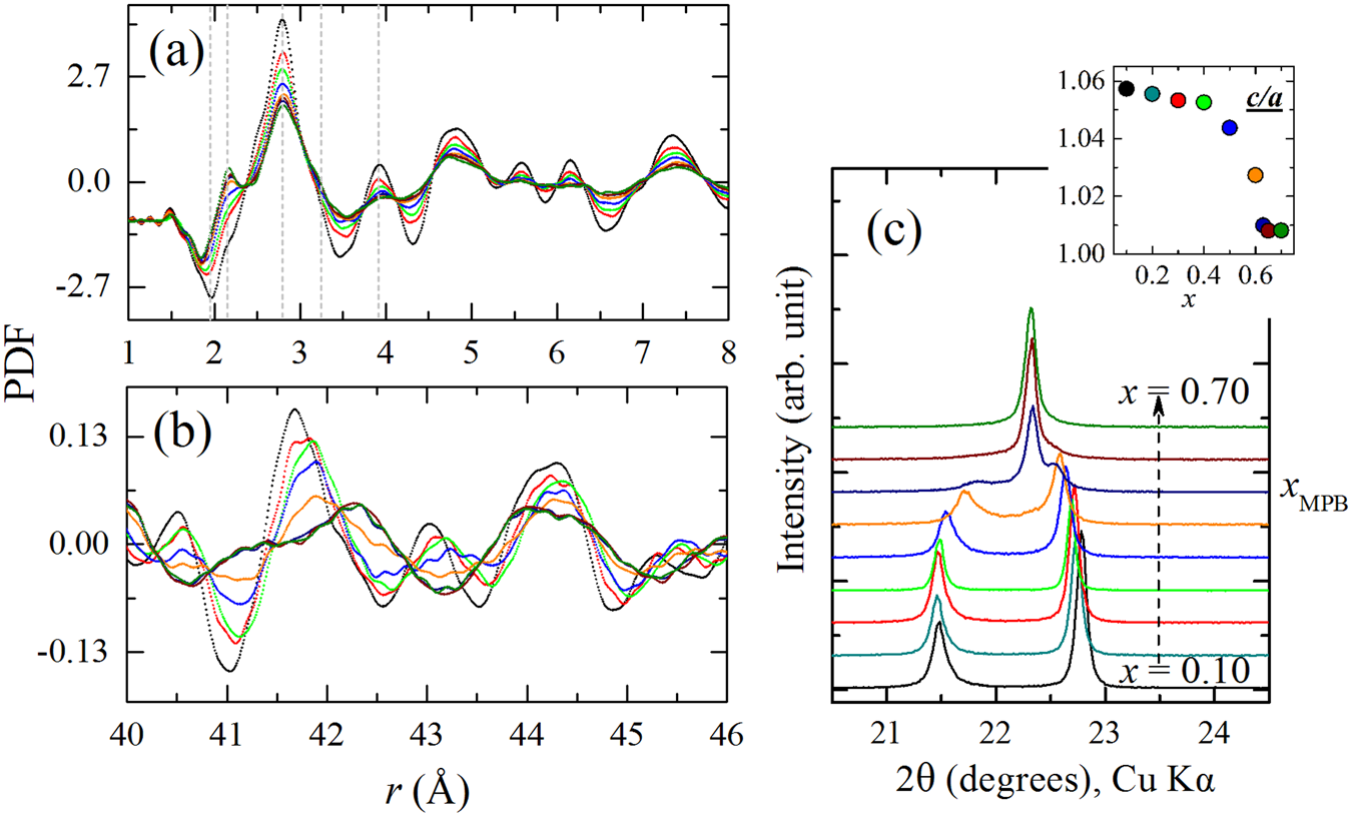
(**a**) Neutron PDFs at ambient conditions in the range 1–8 Å, showing the evolution of short-range atom-atom correlations as a function of composition (*x* = 0.10, 0.30, 0.40, 0.50, 0.60, 0.63, 0.65 and 0.70). The colour scheme representing the composition should be followed from (**c**). The negative peak around 1.9 Å is associated with the nearest-neighbour Ti-O distances, whereas the positive peak standing out at 2.1 Å with increasing *x* corresponds to the first-neighbour Mg-O distances. The peak around 2.8 Å is related to both A-O and O-O nearest-neighbour distances, whereas the shoulder near 3.2 Å refers to first-neighbour A-B distances. First neighbour A-A as well as B-B distances are around 3.9 Å. (**b**) Long-range atomic correlations in the range 40–46 Å, reflecting mainly the changes in the average structure. (**c**) Development of the {001}_*pc*_ Bragg peak with *x* from room-temperature XRD data. The composition-induced change in the average structure is apparent: the strong tetragonal splitting diminishes near the MPB, leading to a single peak at *x* = 0.70. The inset shows the ratio between the unit cell parameters *c*/*a* refined in a tetragonal metrics, which is indicative of the average strain of the system. The entire 2 *θ* range of the laboratory XRD pattern and the x-ray pair distribution functions obtained from synchrotron XRD (APS facility at the Argonne National Laboratory) data as a function of composition can be found in the supplementary information (Figs S2 and S3).

Figure 1(a and b) shows the development of the PDFs for *x*BMT-PT as a function of composition derived from the neutron total scattering data along with the {001}_*pc*_ (pc refers to the pseudocubic setting) Bragg peaks extracted from in-house XRD data (Fig. 1c). The long-range correlations in the PDFs as shown in Fig. 1b in the range 40–46 Å exhibit a distinct change at the MPB composition, which can be easily linked to the observed trend in the Bragg peaks with *x*. However, the short-range correlations up to 8 Å, where peaks can be uniquely associated with the different first-neighbour atom-atom distances in the perovskite-type structure, do not imply for an abrupt change to single out the MPB composition (Fig. 1a). Nevertheless, it appears that the peaks of the PDF at *x* = 0.10 are most pronounced and then it gradually broadens with increasing *x*. The evident gradual changes continues up to the MPB composition and become almost negligible for the compositions *x* = 0.63, 0.65 and 0.70 in the distances longer than approximately 5.1 Å. The changes seen in the peak shape with increasing *x*, suggest enhanced structural disorder, which indicates the composition-induced structural phase transition is more of a order-disorder type than a displacive type. This distinction from a pure displacive-type structural phase transition is an important tag for a ferroelectric system and has impact on the properties especially under external stimuli and non-ambient conditions.

**Figure 2:**
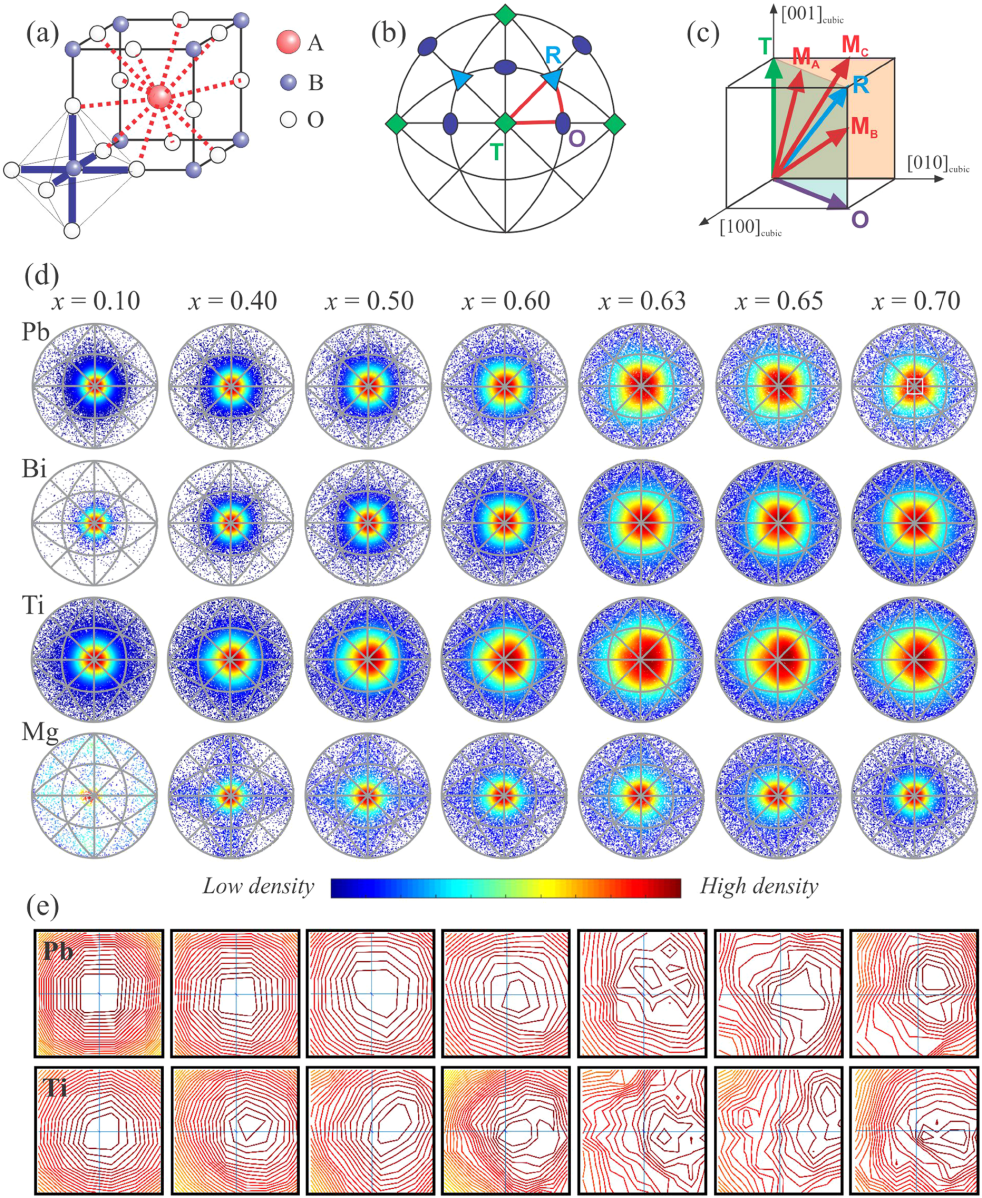
(**a**) Sketch of the aristotype perovskite structure, showing the distinct oxygen environment for the A-site and the B-site cations. (**b**) Typical stereographic projection of a cubic system along the [001] direction. T, R, and O stand for tetragonal, rhombohedral and orthorhombic, respectively, and the symbols in the stereograph represent the preferred directions of cationic displacements in the corresponding polar crystal classes. The red lines mark the crystallographic planes of possible monoclinic polar classes. (**c**) Net polarization-﻿directions﻿ in a perovskite-type structure in the case of tetragonal (T), rhombohedral (R), orthothombic (O), and monoclinic (M_*A*_, M_*B*_, M_*C*_) distortion. M_*A*_ and M_*B*_ correspond to *Cm* with the polarization vector along [*uuv*]_*pc*_ with *u* < *v* and *u* > *v*, whereas M_*C*_ denotes *Pm* with the polarization vector along [0*uv*]_*pc*_^61^. (**d**) [001]_*pc*_ stereographs for the cation shifts as determined from the refined RMC models. Results from 20 independent runs have been combined to generate good statistics. The colour of each point was assigned following the density distribution around that point. (**e**) Zoomed-in central regions of the stereographs by a factor of 10. For example, the magnified area is marked by a white square in the stereograph of Pb for *x* = 0.70. The contour lines are obviously the isolines of density.

In order to extract more specific and quantitative information about the local structural changes across the MPB, we have carried out typical big-box modelling based on the reverse Monte Carlo (RMC) method against the experimental PDF. The analyses were restricted to the atomic-distance range 1–20 Å to ensure that the resulting model solely reveals the local correlations^38, 39^. Very recently, there have been a few reports on popular ferroelectric systems containing Pb and/or Bi using similar big-box modelling applying RMC technique, which have shown how the local structural diversity could be related to the anomalous macroscopic properties^32, 34, 36, 37, 40^. A similar method, first described by Keeble *et al*.^35^, was adopted here to map the distribution of the cationic displacement directions with respect to the centre of their corresponding oxygen polyhedra on a stereograph. These graphs essentially help to evaluate the behaviour of the individual cations with *x*. The magnitude and the direction of the polar displacements of the cations are both crucial for a ferroelectric material since it affects the polarization as well as the structure of the system, and consequently asserts the ensuing macroscopic properties^6^. Figure 2 shows such stereographs for different cations as a function of *x*. For both Pb and Bi, it is apparent that at low values of *x* the directions of off-centre displacements are consistently along the [001]_*pc*_ direction (tetragonal distortion) with a gradual dispersion of the high-density region as *x* increases. In addition, an abrupt enlargement of the dispersion can be seen particularly for the A-site cations from *x* = 0.60 to 0.63 (see Fig. 2d). The strong coupled behaviour of the A-site cations could be justified with their similar electronic properties, however it should be noted that neutron scattering cannot distinguish well between Pb and Bi as they have very similar scattering lengths. Although Ti seems to follow the A-site cations with the composition, the change in the direction-distribution is however smoother, with a relatively higher level of dispersion on the stereographs for all *x* compared to that of the A-site cations. Mg seems to behave uniquely as it tends to scatter initially on the {001}_*pc*_ planes. However for *x* ≥ 0.50 that preference becomes weaker, and at *x* = 0.70, the Mg shifts are predominantly along the [001]_*pc*_.

Apart from these direct observations, the maps can also help to derive further characteristics of the local structure. On the basis of the density distribution of the points in Fig. 2, it is apparent that the inclusion of Bi^3+^ and Mg^2+^ in PbTiO _3_ triggers a structural instability at the local level, and the system is evolving from a strongly anisotropic to a rather isotropic in terms of the displacement directions of the individual cations, which could equally be seen as a reduction of the energy difference between the different orientation-states with the increase of *x*. In addition, this statistical variation of directions with *x* suggests the system gradually becomes more pliable to accommodate non-collinear polar shifts of the cations while approaching MPB from the tetragonal side. This feature in general has direct consequences on the properties of a system, where polarization is coupled to strain and the strain is considered as a primary constraint to the cooperative switching of the dipoles under external fields^41^. Altogether this can be seen as a direct observation of a thermodynamical picture where the individual potential functions for cations are flattened, which in turn provides greater flexibility for the local polarization^6, 42^.

Considering the densities of the favoured directions for *x* ≥ *x*_MPB_, the maps cannot refer a specific average symmetry for the system. Although the x-ray powder diffraction results suggest a single monoclinic *Pm* phase above the MPB (*x* = 0.70), where the polarization vector should remain preferably within the {100}_*pc*_ planes (see Fig. 2c), it is inconclusive from the gross trend in Fig. 2d. However, the zoomed-in central regions of the stereographs around [001]_*pc*_ for Pb and Ti (see Fig. 2e, Bi shows the same trend as Pb) further reveal that there is a subtle yet distinct difference in the direction-trends on a local scale. In particular, Ti exhibits an inclination to the [111]_*pc*_-type polarization even at *x* = 0.10 in comparison to the A-site polar displacements. The A-site cation polar shifts exhibits a deviation in tendency from [001]_*pc*_ to [111]_*pc*_ in the range 0.30 ≤ *x* ≤ 0.60, and beyond that it is highly degenerate. However at *x* = 0.70, there is a weak preference of staying on the {110}_*pc*_ plane, whereas Ti polar displacements shows a tendency to be on the {100}_*pc*_ plane. This as a whole, can be related to all types of monoclinic distortions (M_*A*_, M_*B*_ and M_*C*_), commonly considered for ferroelectric systems based on the perovskite structure. Nevertheless, this emphasizes the fact that the local structural disorder is greatly enhanced with large values of *x* for the family of *x*BiMeO_3_-(1 − *x*)PbTiO_3_, where the Bi-containing end members are mostly not stable at ambient conditions. As a result, conventional analyses of the powder diffraction data through the Rietveld method often face challenges in providing unambiguous average structural description particularly for *x* ≥ *x*_MPB_^36^.

**Figure 3:**
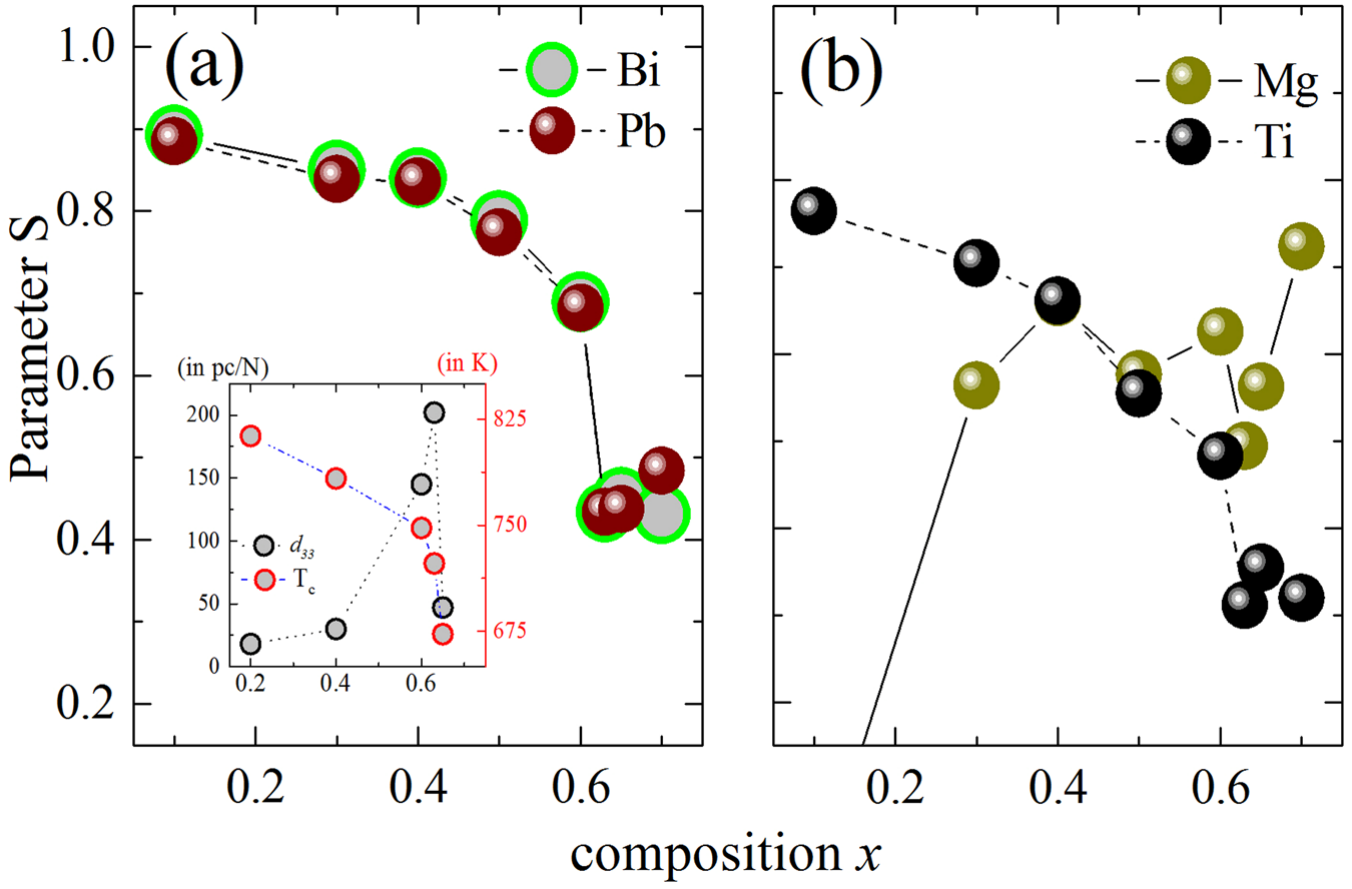
Orientation order parameter S = 1.5〈cos^2^ *θ*〉 − 0.5 showing the degree of deviation of the direction of the cationic off-centre displacements from the [001]_*pc*_ direction. S gradually decreases in the range *x* = 0.1 − 0.5 for the A-site cations and Ti, however from *x* = 0.50 it suddenly drops on the approach to the MPB at *x* = 0.63. Ti shows comparatively lower values of S, meaning a higher level of disorders. The inset shows the variation of the room-temperature piezoelectric coefficient *d*_33_ and the Curie temperatures as a function of composition^23, 25^. The apparent link between these physical parameters and the observed structural behaviour has been discussed in the text.

In order to quantify the observed distribution of the points on the stereographs, we have used a common orientation order parameter S = (1.5〈*cos* ^2^ *θ*〉 − 0.5)^43^, where *θ* represents the angle between the cation displacement direction and the [001]_*pc*_ direction. The cosine is averaged over all displacement directions, and weighted by the corresponding density from the stereographs. The variation of values of S in Fig. 3 depicts the sequence of transition with *x* by a measure of ordering of the cations with respect to our chosen direction [001]_*pc*_, and intriguingly, it reaches its minimum value of around 0.4 at *x*_MPB_ for all ferroelectrically active cations. This provides an unequivocal evidence that the system acquires a state with maximum structural instability at the MPB, where the piezoelectric coefficient *d*_33_ reaches its maximum value, while the Curie temperature *T*_*c*_ abruptly decreases.

**Figure 4:**
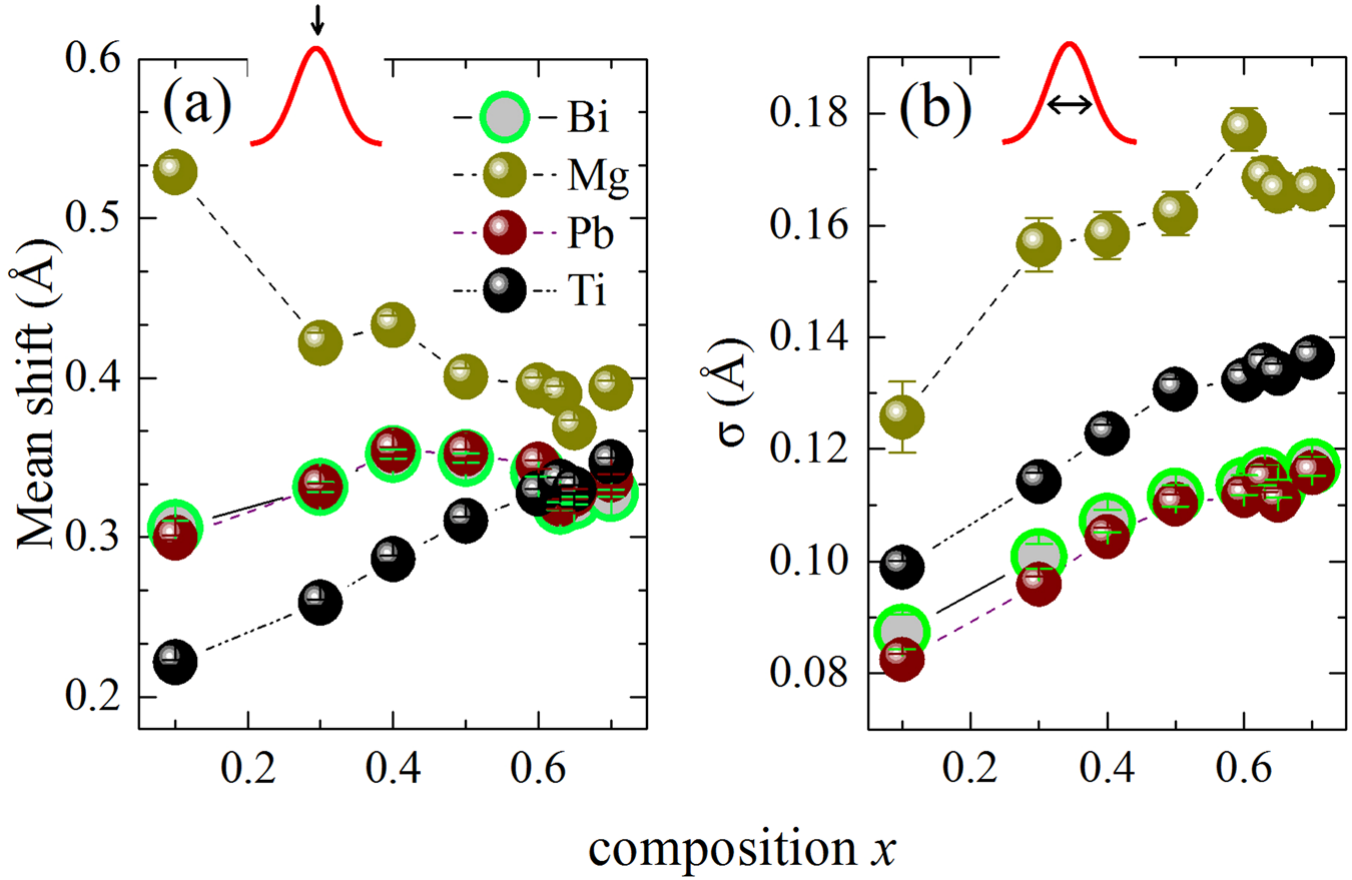
Mean magnitudes and standard deviations *σ* of the cationic polar displacements determined from the centre of respective oxygen polyhedron as a function of composition.

Figure 4 demonstrates the mean values of the polar shifts of the cations with *x* as determined from the refined structural model, which can be directly related to the values of intrinsic polarization of the system. The shifts are comparable with the reported values based on experiments as well as theoretical calculations on several similar Pb and Bi containing solid solutions^44–46^. Apart from Mg, cations show a gradual increase in the average shifts with increasing *x*. However, this increase cannot account for the abrupt rise of the *d*_33_ coefficient at the MPB. This is highly significant, because it indirectly underlines the critical influence of enhanced randomness of the shift-directions on the response functions driven by an external stimulus. Moreover, it reinstates the fact that the ferroelectric Curie temperature is heavily coupled to the microscopic strain of the system, and hardly influenced by the magnitudes of polar shifts of the cations. It is further interesting to note that the mean shifts become almost equal in magnitude for the A-site and the Ti cations on the onset of the MPB, which characterise competing A- and B-site driven ferroelectricity^47, 48^. The magnitudes of the displacements and the standard deviations for the Mg are unexpectedly large considering the chemical behaviour of the cation^24, 46, 49^. For low values of *x ﻿﻿﻿however﻿﻿*, bigger uncertainty can be expected in Mg-O distances, especially because the Mg-O peak sits on the shoulder between the negative Ti-O and the positive Pb/Bi-O peak (see Fig. 1a). However as the *x* increases, the respective mean shifts seem to become smaller gradually and therefore the values should be more reliable.

### Raman scattering analysis

**Figure 5:**
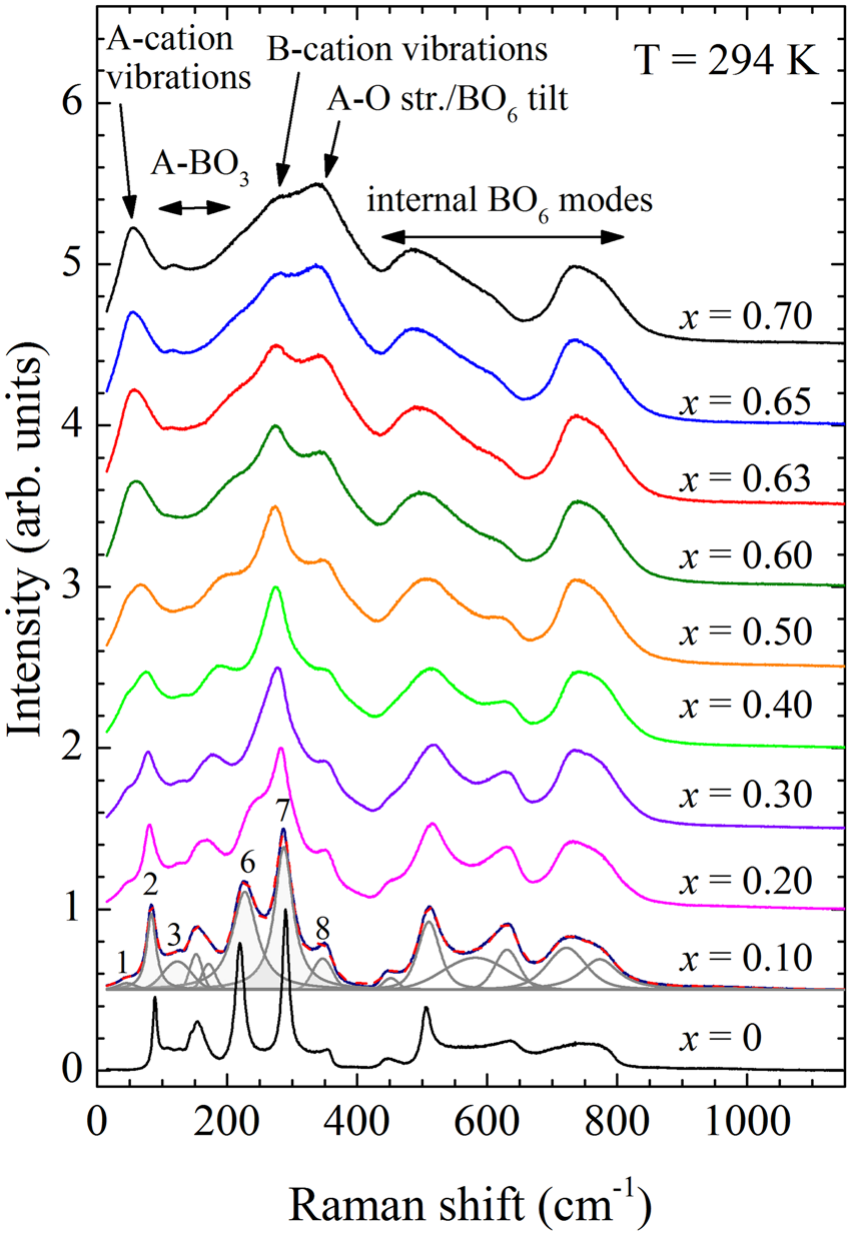
Room-temperature Raman spectra of *x*BMT-PT along with the model pseudo-Voigt functions and the resultant curve (red dashed line) for *x* = 0.10. Peak assignment is based on the comparison with other complex perovskite-type ferroelectrics^50, 51^.

**Figure 6:**
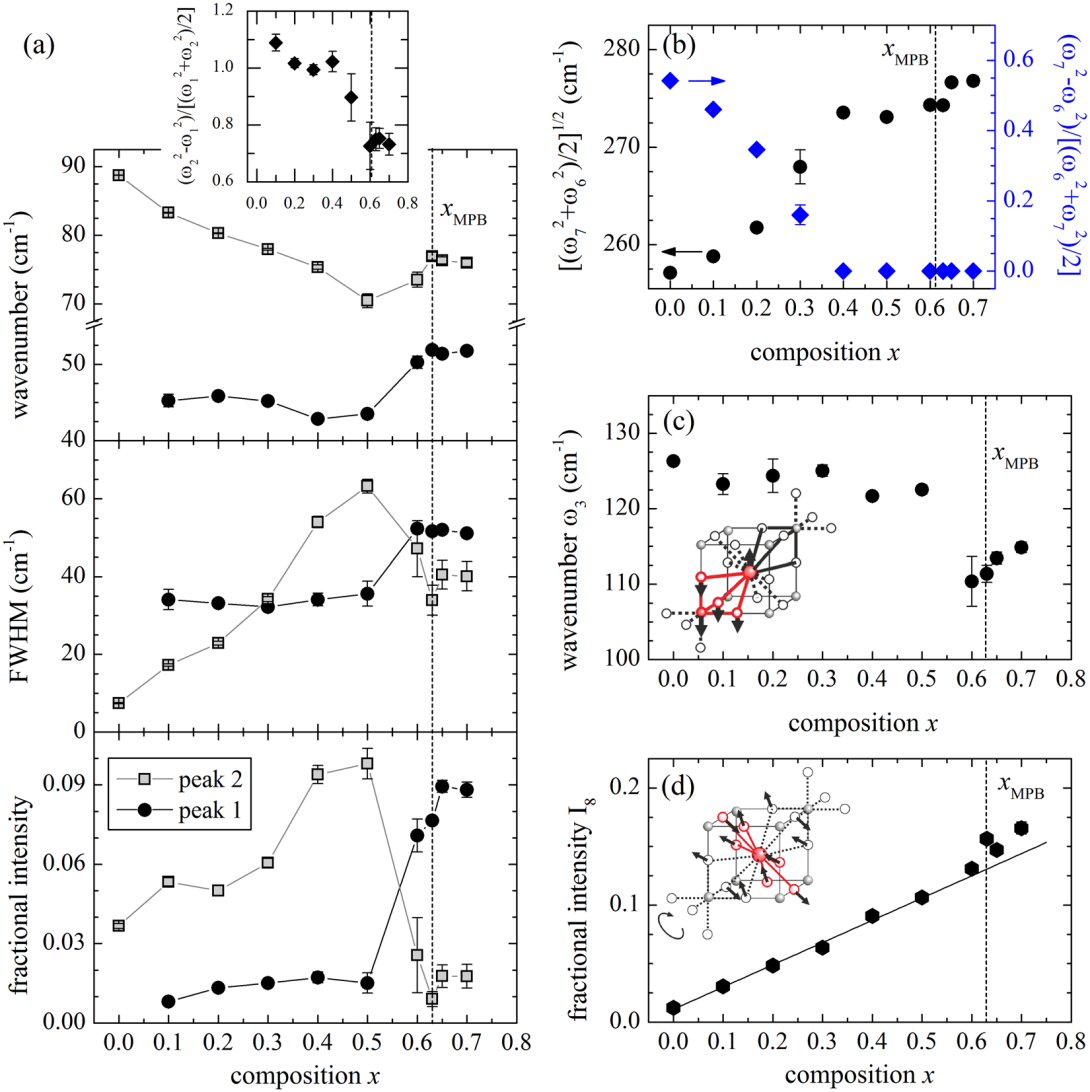
Composition dependence of (**a**) the phonon modes dominated by A-site cationic vibrations (peaks 1 and 2), (**b**) the average squared wavenumber and splitting of phonon modes dominated by B-site cationic vibrations (peaks 6 and 7), (**c**) the wavenumber of peak 3 generated by BO_3_ vibrations against A-site cation vibrations (the inset represents the atomic vector displacements in the aristotype cubic structure), (**d**) the intensity of peak 8 corresponding to A-O stretching vibrations, which can also be thought as BO_6_ tilting vibrations (the inset represents the atomic vector displacements in the prototype cubic structure). Peak numbering are as given in Fig. 5. The dashed lines mark the MPB. The solid lines in (**a**) are merely guides for the eye; the solid line in (**d**) is a linear fit to data points with *x* between 0 and 0.5.

Figure 5 shows the composition-dependent Raman spectra for the *x*BMT-PT system with the corresponding assignment of different modes in complex perovskite-type ferroelectrics^50, 51^. It is evident that there is a gradual change in the spectra as a function of *x* and therefore, it is difficult to pin-point the MPB intuitively. However the detailed analysis of the individual spectra reveals distinct anomalies for several phonon modes. The lowest energy modes near 44 and 88 cm^−1^ (peaks 1 and 2), which are commonly dominated by A-site cationic vibrations^50–52^, soften around *x* = 0.4 − 0.5 < *x*_MPB_ (see Fig. 6a). In particular peak 2, which is observed in undoped PbTiO_3_, shows a distinct minimum in the wavenumber vs *x* together with a maximum in FWHM vs *x*. This provides a clear evidence for a local-scale structural transformation driven by a rearrangement of heavy A-site cations^18^.

It is worth noting that in single crystals of PbTiO_3_, the Raman scattering near 88 cm^−1^ corresponds to the E(TO) soft mode. Polycrystalline ferroelectric solid solutions however possess oblique phonon states of mixed character. Hence, a direct ‘one-to-one’ assignment to a certain irreducible representation is not appropriate. Nevertheless, the *x* value at which peak 2 softens, matches the composition exactly at which *σ* seems to reach a level of saturation for all ferroelectrically-active cations (see Fig. 4), on the onset of an enhanced orientation disorder in polar shifts of the cations (see Fig. 3).

The Raman scattering at 44 cm^−1^ (peak 1) appears only in the presence of BMT. However, it cannot result exclusively from Bi vibrations developing a two-mode behaviour of the peak around 88 cm^−1^, because if that would be the case, then the intensities of both peaks would vary gradually with *x* ^53^. This is evidently not the case here (see Fig. 6a), and indicates that the new peak should be related to a different structural state of A-site cations rather than to a different chemical species. In fact, the intensity of peak 1 abruptly increases between *x* = 0.5 and *x*_MPB_, along with a sharp hike in the peak width. Altogether this affirms that the addition of BMT induces a distinct energetically favourable state in the A-site cation subsystem, which becomes dominant at *x* ≥ *x*_MPB_. Besides, larger peak widths at compositions *x* ≥ *x*_MPB_ for both peak 1 and 2 indicate strong structural disorder, which could be equally conceived as a frustration in the coupling of coherent local distortions. This conforms wonderfully to the features of the orientation order parameter S (Fig. 3) and *σ* (Fig. 4b), revealed from the PDF analysis. The occurrence of the MPB is actually mirrored by the A-cation dynamics following the fact that the squared wavenumber difference reaches its minimum value (see the inset in Fig. 6a), i.e. the difference between the two energy states becomes minimal precisely at the MPB.

The phonon modes around 230 (peak 6) and 280 cm^−1^ (peak 7) are related to the B-site cationic vibrations^50–52^. These two modes evidently merge at *x* = 0.4, which could be related to the suppression of the prominent tetragonal distortion within the BO_6_ octahedra. It is important to note that at *x* = 0.4 the mean polar shifts of the A-site cations get to its maximum value but the Ti polar displacements continue to increase and become almost equal in magnitude with those of the A-site cations exactly at the MPB. This holds a favourable condition to constitute a strong coupling between the off-centred A- and B-site cations. Evidently, the phonon mode involving the BO_3_ vibrations against the A-site cation vibrations has a well pronounced minimum at *x*_MPB_ (see Fig. 6c), which apparently drives the system to a phase transition.

The Raman peak near 350 cm^−1^ increases steadily in intensity with the increase in *x*, and it has even higher intensity than the B-cation vibration mode near 280 cm^−1^ for compositions above the MPB (see Fig. 4). Lattice-dynamics calculations for Pb-based complex perovskite-type oxides reveal that the Raman scattering near 350 cm^−1^ arises from a point phonon mode, which is silent (T_2*u*_ symmetry) in the aristotype structure $Pm\bar{3}m$ but may generate Raman intensity in a distorted double-perovskite structure^54^. The doubling of the unit cell may be induced by NaCl-type local chemical order at the B site and/or antiferrodistortive structural order, but only the latter may generate Raman activity of the phonon states near 350 cm^−1^. The inspection of the atomic vector displacements indicates that this mode is comprised of oxygen vibrations along the A-O bonds in the {111}_*pc*_ planes, but it can be also thought as a rotation of the BO_6_ octahedra about the 〈111〉_*pc*_ directions (see inset in Fig. 6d). Consequently, the intensity of the Raman peak near 350 cm^−1^ is expected to increase with the development of the BO_6_ tilts. This was in fact detected by combined Raman scattering and neutron/synchrotron x-ray diffraction in a few relaxor ferroelectrics under high pressure^55–57^. Therefore, the gradual increase in the intensity of the phonon mode near 350 cm^−1^ with increasing *x* suggests that the dynamic BO_6_ tilting i.e. the antiferrodistortive ordering becomes significant for large values of *x*. A further crosscheck of this fact was found in the analysis of the RMC refined structural models where a gradual decrease of the B-O-B bond angle, which is typically used as a measure of the static tilts of the BO_6_ octahedra^58^, was recorded with the increase in *x* (see Supplementary Figure S5). Hence this ensuing development of local dynamic antiferrodistortive order upon doping can effectively interfere with the cationic polar shifts through suppressing the flexibility and the affinity to reorient under external field.

## Summary and Implications

On the whole, our results provide an atomistic view of the development of composition-driven structural phase transition of a ferroelectric solid solution based on the perovskite structure with chemical disorder on both A and B sites, based on a combined analyses of the PDF and Raman scattering data. It is noted that the apparent change in the average structure with increasing content of BMT can be envisaged essentially as concurrent increase in local structural disorder in terms of off-centre static displacements of cations and randomness in their directions. However, it is the degree of stochasticity of the polar-shift directions (parameter S) which evolves systematically with composition and describes the development of structural instability, leading to the morphotropic phase boundary. But this parameter alone cannot explain the structure-property connections. We found that at MPB, all ferroelectrically active cations (A-site Pb^2+^ and Bi^3+^, B-site Ti^4+^) acquire very similar off-centre displacements, which together with the enhanced flexibility lead to a strong dynamic coupling between the polar shifts of the A- and B-site cations. This suggested that the combination of such structural instability and dynamic coupling is pivotal and an absence of any one of these factors may not bring about the expected boom in the properties, following the fact that the abrupt fall in the piezoelectric coefficient perfectly correlates with the diminished coupling factor for *x* > *x*_MPB_. These specific aspects of atomic-level structural correlations have not been conceived so far for a ferroelectric system, and should be however distinguished from the random orientation of the so-called polar nanoregions or local electric fields, commonly attributed to relaxor ferroelectrics. Our refined structural models did not suggest any notable clustering or chemical ordering at the A- or B-sites in support of that (See Supplementary Figure S7). The present random-direction model does conforms to the concept of polarization rotation or polarization extension but further put forward the necessity of the collaborative dynamic coupling effect, which should be an important part of the atomistic driving force for the enhanced properties often seen around an MPB.

Generally speaking, the proposed model featuring distinct static and dynamic characteristics might be applicable for a broad range of perovskite-based ferroelectric solid solutions especially with Pb and Bi where similar behaviour of the average structure have already been reported. However it entreats on a number of colligated issues, such as the distinction of the composition-driven phase boundaries from the temperature or pressure-induced phase boundaries, nature of the B-site chemical disorder (here Mg acts as a modifier of the coupling processes between the ferroelectrically active elements), combination of ferroelectric-antiferroelectric order (as in PZT), and especially the MPBs of Pb-free systems in order to develop efficient design rules.

## Methods

### Samples

Ceramic samples of *x*BMT-PT with *x* = 0.10, 0.20, 0.30, 0.40, 0.50, 0.63, 0.65, and 0.70 were prepared following the conventional solid-state synthesis route, details of which can be found elsewhere^25^. Room-temperature powder x-ray diffraction data (Stoe Stadi-MP powder X-ray diffractometer) were collected to verify the formation of a single perovskite phase. Electron microprobe analyses (wavelength-dispersive Cameca Microbeam SX100 SEM-system) averaging over 50 points from each compound were conducted to confirm the expected chemical compositions (see Supplementary Material Fig. S1). Commercially available powder of PbTO_3_ (Sigma Aldrich, purity ~99.9%) was used as a reference sample in Raman spectroscopic analyses.

### Total neutron scattering and RMC modelling

Room-temperature neutron total scattering data were collected at the Nanoscale Ordered Materials Diffractometer (NOMAD) at the Spallation Neutron Source of Oak Ridge National Laboratory. NOMAD is a dedicated instrument for the total scattering experiments and allows to collect data for a wide range of reciprocal-space vector Q (=4*π* sin*θ*/*λ*), which is a necessary condition to produce reliable PDFs. For our measurements the Fourier transformations were done with Q_*max*_ = 31.4 Å^−1^, which provided a real-space resolution of around 0.1 Å.

RMC modelling of the structure against the PDF data was performed using the RMCprofile package^39^. The starting models for different compositions were built using the structural parameters determined from prior Rietveld refinements of the neutron powder diffraction pattern. The modeling box size was approximately 54 × 54 × 54 Å^3^ and consisted of ~13000 atoms. There were 20 independent runs for each composition in order to have good statistics of the structural parameters. The DISCUS software^59^ was used to extract the various structural parameters from the refined models.

### Raman scattering

Raman spectra were collected with a Horiba T64000 triple-grating spectrometer equipped with an Olympus B41 confocal microscope (50x objective), and a Symphony liquid-N_2_-cooled CCD detector. The spectra were recorded with a laser wavelength of 514.5 nm, on plate-shaped pellets in backscattering geometry, with a spectral resolution of ~2 cm^−1^, and peak-position precision of 0.35 cm^−1^. No polarization, orientation, and spatial dependence of the Raman spectra were detected. The measured spectra were temperature-reduced to account for the Bose-Einstein phonon occupation factor and fitted with pseudo-Voigt (*PV*) functions (*PV* = *q* * *Lorentz* + (1 − *q*) * *Gauss*) to determine the peak positions, full widths at half maximum (FWHMs), and integrated intensities. The criterion for the maximum number of fitted peaks was *dI*/*I* < 0.5 for all peaks, where *I* and *dI* are the calculated integrated intensity and the corresponding uncertainty, respectively^60^. In fact, for all compounds the achieved ratios *dI*/*I* were less than 0.25.

## Electronic supplementary material

Supplementary Informations
