# Supplementary material for: Atomic-level structural correlations across the morphotropic phase boundary of a ferroelectric solid solution: xBiMg1/2Ti1/2O3-(1 − x)PbTiO3
Source: Sci Rep. 2017 Mar 28;7:471. doi: 10.1038/s41598-017-00530-z (PMC5428731; doi:10.1038/s41598-017-00530-z)
Supplement: Supplementary file 1 — Supplementary Informations [file 41598_2017_530_MOESM1_ESM.pdf]

Atomic-level structural correlations across the morphotropic phase boundary of a ferroelectric solid solution: xBiMg$_{1/2}$Ti$_{1/2}$O$_3$-(1-x)PbTiO$_3$

Kaustuv Datta[1,*], Reinhard B. Neder[2], Jun Chen[3], Joerg C. Neuefeind[4], and Boriana Mihailova[1]

[1]Department of Earth Sciences, University Hamburg, Hamburg - 20146, Germany.
[2]Department of Crystallography and Structure Physics, University of Erlangen-Nürnberg, Staudtstraße 3, Erlangen -
91058, Germany.
[3]School of Metallurgical and Ecological Engineering, University of Science and Technology Beijing, China.
[4]Chemical and Engineering Materials Division, Oak Ridge National Laboratory, Oak Ridge, Tennessee 37831,
United States.
[*]kaustuv.datta@uni-hamburg.de

Supplementary Figures:

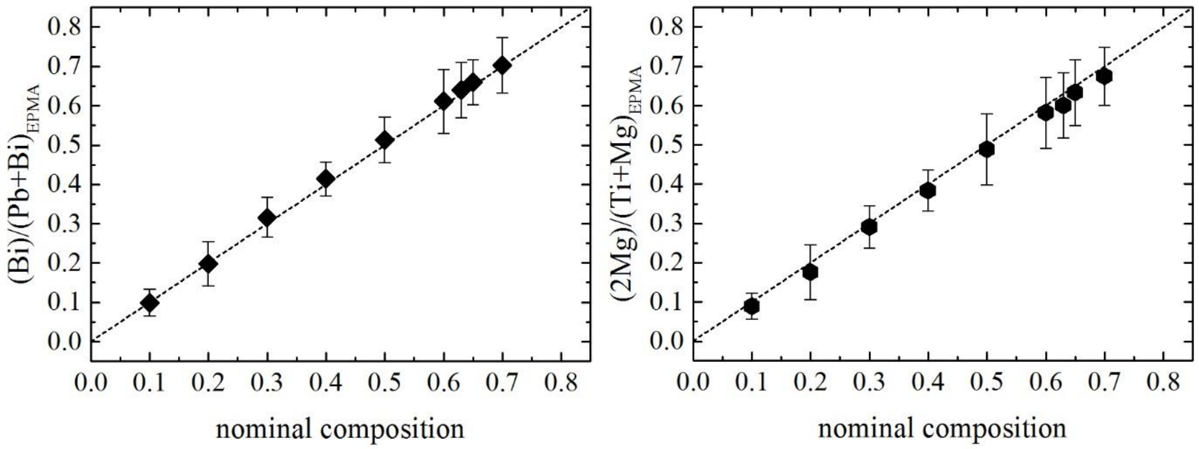

Figure S1: The relative amounts of Bi and Pb as well as of Mg and Ti derived from a wavelength-dispersive electron microprobe analyses performed over 50 points from each compound versus the nominal composition x. The dashes lines represent the one-to-one correlation. As can be seen, the measured mean values of Bi/(Bi+Pb) (diamonds) as well as of 2Mg/(Ti+Mg) (hexagons), representing the A-site and B-site occupancy, perfectly match the expected nominal values; the error bars correspond to the standard deviations.

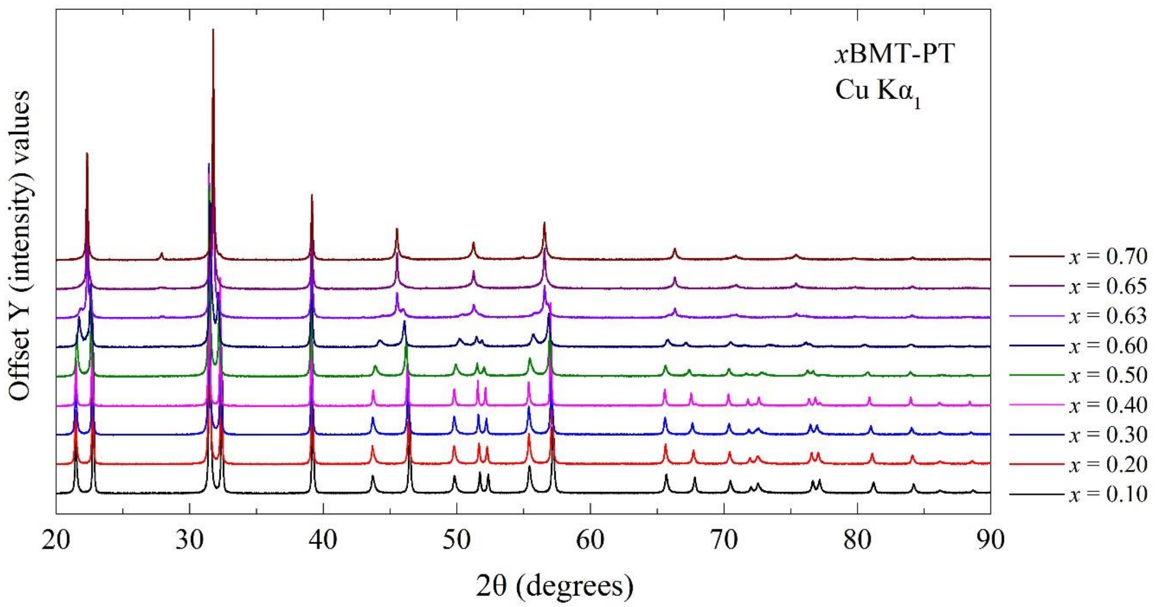

Figure S2: Room temperature x-ray powder diffraction data for the xBMT-PT ceramic samples as a function of composition. The data were collected using a laboratory x-ray diffractometer (Stoe Stadi-MP powder X-ray diffractometer) providing monochromatised Cu $K\alpha_1$ radiation. These patterns are consisted with the previous reports [1] in terms of the average structure and it is evident that x = 0.63 can be assigned as the MPB composition.

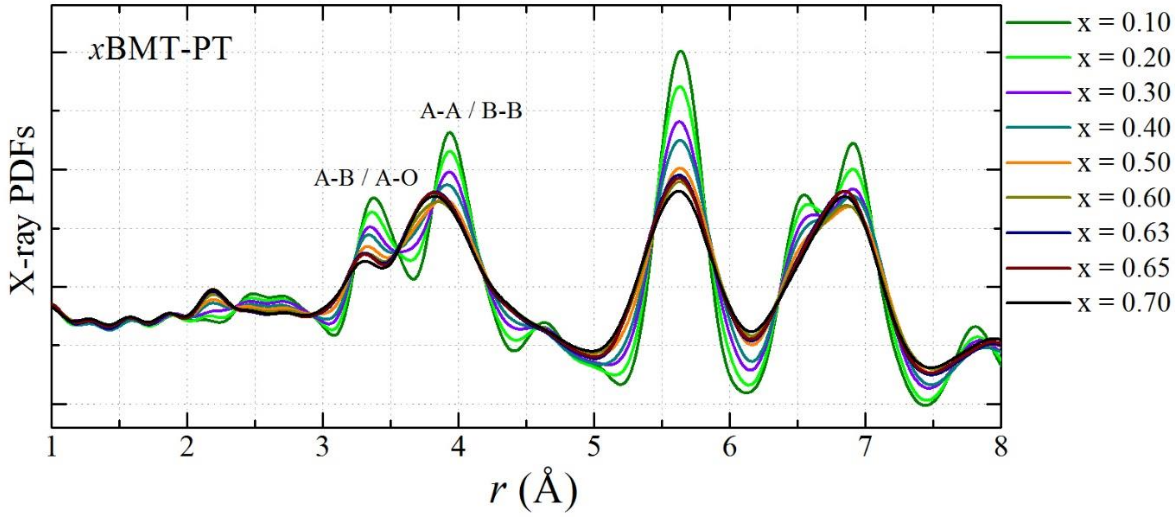

Figure S3: Room temperature x-ray pair distribution functions of xBMT-PT. These complementary data sets were collected at the Argonne National Laboratory using the beamline 11-ID-B of the Advanced Photon Source (APS) facility with the incident beam wavelength of 0.2114 Å. The first significant peaks around 3.3 Å correspond to the A-B and A-O distances, whereas the peak around 3.9 Å corresponds to the A-A and B-B distances for the perovskite-type (ABO_3) structure. It is evident that the range 1.8 - 2.5 Å is heavily affected by the Fourier ripples, and therefore it is difficult to extract any reliable information on the B-O distances. As a whole the x-ray PDFs are mostly weighted by the heavy Pb and Bi cations, and this is why it is important to have the neutron scattering data to be able to determine the exact oxygen surroundings of the individual types of cations which is a key to understand the important structure-property relations in perovskite-based ferroelectric systems.

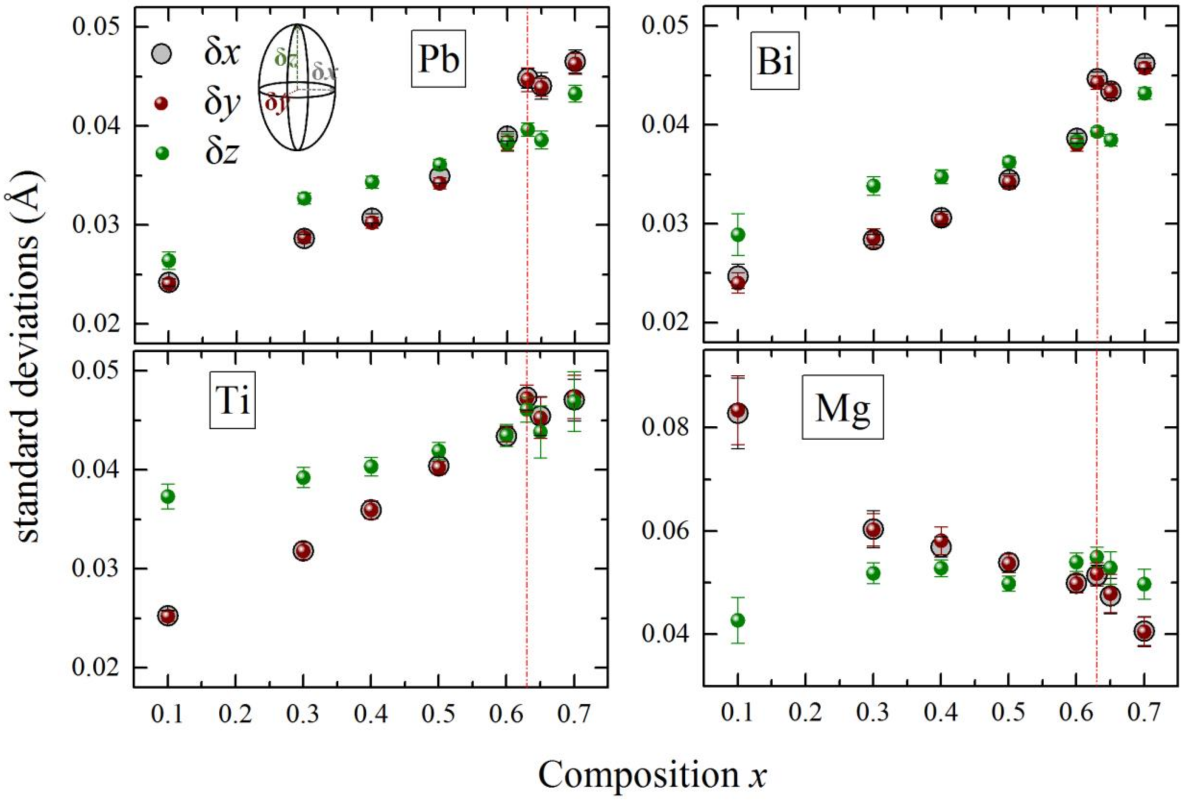

Figure S4: Standard deviations of the x, y and z positions (δ*x*, δ*y* and δ*z*) of the cations as a function of composition, derived from the refined structures. Values from twenty independently refined models were averaged and the errors bars show their dispersion among them. These values can be essentially related to the anisotropic displacement parameters to envisage the displacement ellipsoids of the cations. Evidently, Ti exhibits a distinct gradual reduction of the anisotropy (δ*x* = δ*y* ≠ δ*z*) on the approach to the MPB. The anisotropy for Pb, Bi is weaker than the Ti when $x < x_{MPB}$ and it changes from δ*x* = δ*y* < δz to δ*x* = δ*y* > δz for $x > x_{MPB}$. The trend for the Mg however is opposite in comparison to the rest. The variation of the Ti-anisotropy directly correlates with values of *c/a*, and provides a crosscheck that the RMC-refined model structures are consistent with the average structures determined from the typical Bragg diffraction peak analysis.

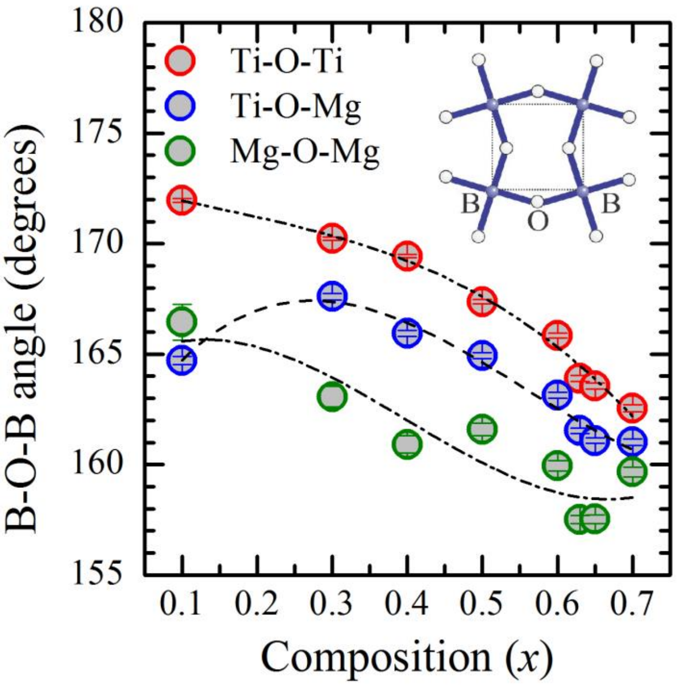

Figure S5: Development of B-O-B bond angle of the perovskite structure (ABO_3) with composition. This angle is particularly sensitive to the static distortions manifested by the tilts of the oxygen octahedra. For an untitled system the B-O-B angle should be ideally 180 degrees, whereas any deviation from that would indicate the occurrence of tilts. The B-O-B angle for three possible combination of cations was calculated for all 20 independent RMC refinements, and then the mean and standard deviation were calculated from their distribution. The trend suggests that tilt distortions are enhanced with increase in *x*.

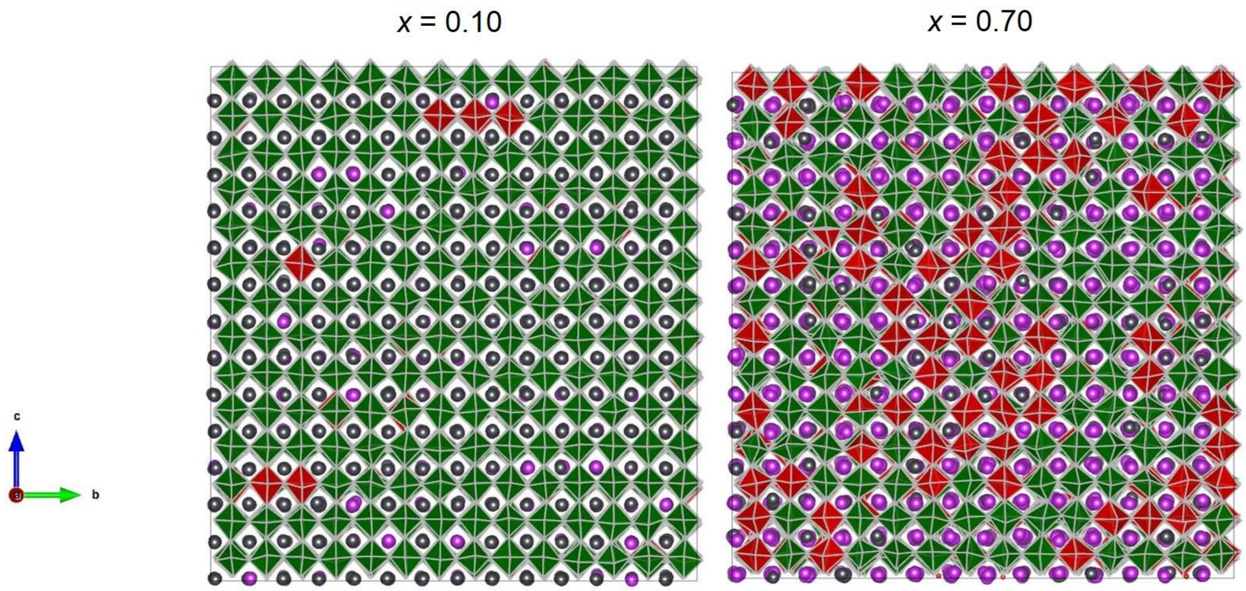

Figure S6: Crystal plots for RMC-refined model structures (*0yz* layer of the configuration) for x = 0.10 and 0.70. It is evident that both Ti (Green) and Mg (Red) octahedras are distorted and manifests tilts. Moreover, as expected, the composition x = 0.70 exhibits visibly higher level of distortions than x = 0.10.

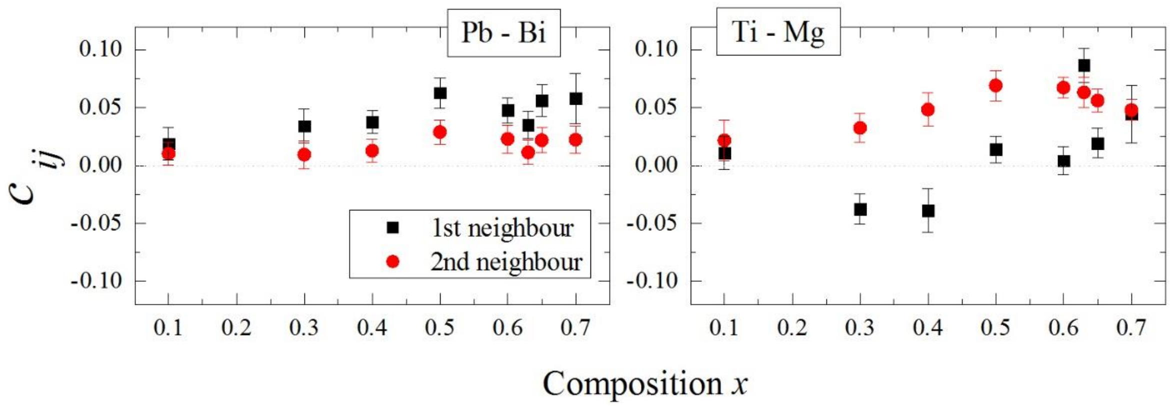

Figure S7: Chemical order parameter c_{ij} with composition x, defined as follows: $c_{ij} = \frac{P_{ij} - \theta^2}{\theta(1-\theta)}$

P_{ij} refers to the total probability of sites *i* and *j* being occupied by the same type of atom and *θ* is the concentration of the cation in the whole system. c_{ij} becomes negative when the two sites *i* and *j* prefer to be filled by different types of atoms, while the positive values indicate the higher likelihood of sites *i* and *j* occupied by the same type of atom [2]. The maximum negative value of c_{ij} for a given concentration can be $-\theta/(1-\theta)$, whereas the maximum positive value possible is +1.

We have calculated the parameter c_{ij} for 20 configurations and then averaged. The values do not indicate for any strong preference for a local chemical ordering either between A-site or B-cite cations. However, comparatively there is a very weak tendency between the B-site cations to form chemically ordered regions when x = 0.30 and 0.40.

Theoretical investigation on the structure of $Bi(Mg_{1/2}Ti_{1/2})O_3$ have speculated on the existence of chemical order in this system [3, 4] and in general, it is often suggested that short-range B-site chemical ordering has a significant role in the composition variations of properties of PZT and other similar systems [5].

References:

[1] C. A. Randall et al. Investigation of a high Tc piezoelectric system: (1-x)$Bi(Mg_{1/2}Ti_{1/2})O_3$–(x)$PbTiO_3$. J. Appl. Phys. 95, 3633–3639 (2004).

[2] T. Proffen et al, Chemical short range order obtained from the atomic pair distribution function. Z. Kristallogr. 217, 47–50 (2002).

[3] K. Miura, M. Kubota, M. Azuma and H. Funakubo, Electronic and Structural Properties of $BiZn_{0.5}Ti_{0.5}O_3$. Jpn. J. Appl. Phys. 48, 09KF05 (2009).

[4] M. Suewattana, D. J. Singh and S. Limpijumnong, Crystal structure and cation off-centering in $Bi(Mg_{1/2}Ti_{1/2})O_3$. Phys. Rev. B 86, 49–52 (2012).

[5] A. M. George, J. Iniguez and L. Bellaiche, Effects of atomic short-range order on the properties of perovskite alloys in their morphotropic phase boundary. Phys. Rev. Lett. **91**, 045504 (2003).
